# Edge-Computing Meshed Wireless Acoustic Sensor Network for Indoor Sound Monitoring

**DOI:** 10.3390/s22187032

**Published:** 2022-09-17

**Authors:** Selene Caro-Via, Ester Vidaña-Vila, Gerardo José Ginovart-Panisello, Carme Martínez-Suquía, Marc Freixes, Rosa Ma Alsina-Pagès

**Affiliations:** GTM—Grup de Recerca en Tecnologies Mèdia, La Salle—Ramon Llull University, 08022 Barcelona, Spain

**Keywords:** wireless acoustic sensor network, low-cost sensor, meshed network, acoustic quality, real-time signal processing, Raspberry Pi, acoustic signal processing

## Abstract

This work presents the design of a wireless acoustic sensor network (WASN) that monitors indoor spaces. The proposed network would enable the acquisition of valuable information on the behavior of the inhabitants of the space. This WASN has been conceived to work in any type of indoor environment, including houses, hospitals, universities or even libraries, where the tracking of people can give relevant insight, with a focus on ambient assisted living environments. The proposed WASN has several priorities and differences compared to the literature: (i) presenting a low-cost flexible sensor able to monitor wide indoor areas; (ii) balance between acoustic quality and microphone cost; and (iii) good communication between nodes to increase the connectivity coverage. A potential application of the proposed network could be the generation of a sound map of a certain location (house, university, offices, etc.) or, in the future, the acoustic detection of events, giving information about the behavior of the inhabitants of the place under study. Each node of the network comprises an omnidirectional microphone and a computation unit, which processes acoustic information locally following the edge-computing paradigm to avoid sending raw data to a cloud server, mainly for privacy and connectivity purposes. Moreover, this work explores the placement of acoustic sensors in a real scenario, following acoustic coverage criteria. The proposed network aims to encourage the use of real-time non-invasive devices to obtain behavioral and environmental information, in order to take decisions in real-time with the minimum intrusiveness in the location under study.

## 1. Introduction

Humans are everyday more used to coexisting with technological devices, even in private environments such as homes. When a home is provided with a set of sensors that enable the automation and monitoring of different day-to-day actions, it can be categorized as a smart home, with a special focus—but not exclusively—on ambient assisted living applications [1]. Nonetheless, the same sensors can be applied in other environments such as private homes, hospitals, universities, students’ residences or nursing homes for different purposes. However, and especially for specific segments of the population, privacy becomes a key issue when accepting those devices [2]. Actually, for a technological system to be accepted, it should be perceived by the users as non-intrusive and non-obtrusive, terms defined in [3]. Mainly, a system is perceived or not as intrusive or obtrusive depending on eight different dimensions: physical dimensions, usability dimensions, privacy dimensions, function dimensions, human interaction dimensions, self-concept dimensions, routine dimensions and sustainability dimensions. In summary, the devices should (i) be of reduced dimensions and be integrated with the architectural aesthetic, (ii) be easy to use, (iii) not reveal more information than the user is willing to share, (iv) not affect the personal relationships of the people using them or make them feel treated differently, (v) not make the people feel anxious about not being able to afford the device in the future.

Most of the users of smart devices believe that their personal raw data gathered by sensors should not be shared with third parties such as ISPs (Internet Services Providers) [4], especially when thinking of sensitive groups, such as old people, children or teenagers. Furthermore, the study in [5] shows the most recent trends in the technical challenges to face attacks and prevent vulnerabilities in networks, especially from an industrial point of view. Based on those requirements and recommendations, and in this regard, the edge computing paradigm has emerged as a powerful tool to perform the signal processing in the sensing nodes to avoid unnecessary transmissions of sensitive raw data. Edge computing refers to moving the computation load and/or storage of data of a system closer to the source in which the data is generated [6]. In this sense, this computation is performed in edge devices, which are composed of computing units connected to sensors that gather data. Besides other advantages, such as faster response times or bandwidth cost savings (achieved by sending processed data instead of raw data), data safety and privacy are also key issues to be taken into account when deciding to use an edge-computing architecture in front of a cloud-based system.

From a communication point of view, the edge nodes may use wired (such as Ethernet) or wireless (such as WiFi or 2G, 3G, 4G or 5G cellular networks) communication protocols depending on the intrinsic characteristics of the scenario in which they would be deployed and the desired features of the network [7]. Another parameter to consider is the amount of data that has to be sent in order to integrate the information required in the central node; this volume of information changes depending on the application, the size of the network and the final goal of the evaluation of the acoustic data collected and processed. These features also involve the desired speed, the distance between nodes, the number of supported devices, the covering area of near technologies (e.g., cellular antennas) and the budget constraints, among others, in order to design the best possible network to satisfy the requirements of the problem under study.

Following this paradigm, this paper presents a meshed Wireless Acoustic Sensor Network (WASN), which consists of a network composed of microphones (i.e., the sensors of the edge devices) and computing units spatially distributed over an indoor environment, capable of monitoring noise levels and detecting certain predefined acoustic events. Specifically, the paper covers the conception of the network since selection and set-up of the hardware of the sensing nodes (including the selection and the calibration of the microphones) and the wireless communication protocol between them, to the comparison of different models of 3D printed boxes to protect the computing units of each node while preventing overheating. The proposed system should fit any indoor environment that aims to measure noise levels or detect acoustic events.

One can find several contributions in the literature focused on the design of low-cost acoustic sensor networks to solve several different requirements, depending on their application. Those contributions can range from a simple WASN design [8], to a system already capable of executing, in real-time, several algorithms and uploading the results in the cloud [9]. The goal of the proposal presented in this paper, which clearly differs from other proposals in the literature, is to obtain the most precise acoustic sensor network with the lowest possible cost, assuming that precision, in this stage, stands for both the precision of acoustic measurements and future acoustic event detection algorithms. For this purpose, the requirements of both data collection hardware design and computational capabilities to develop signal processing algorithms have been considered. The precise comparison of our approach with regards to other proposals in the literature is widely discussed and detailed in the Discussion section of this paper (Section 6). Focusing more on the details and the proper contributions of this work, the most relevant issues presented in this paper are:

(1) The design of a general-purpose Wireless Acoustic Sensor Network, focusing on the design of the sensing nodes using a commercial computing unit and microphone. Different commercial computing units have been evaluated in terms of cost and capabilities. The selected computing unit is capable of performing edge-computing calculations, which enables acoustic signal processing to be carried out in the sensor without the need to send raw acoustic data to the cloud. Regarding the microphone, three different options have been evaluated in terms of cost, linearity and calibration capabilities. The plug-and-play selected option enables omnidirectional data to be obtained without the need for an external Analogue to Digital Converter.
(2) The conception of a wireless meshed network topology to communicate the sensing devices in a Local Area Network (LAN), maximizing the coverage area of the network without using external hardware such as a router or a range extender. For this purpose, a real-world test has been carried out in a scenario composed of three rooms and one terrace, with a long distance among nodes. A comparison between using a direct connection from nodes to a central unit or a meshed topology has shown that the latter option enables us to drastically reducing the number of frames lost during the experiments.
(3) The design of a coverage (i.e., a protecting 3D-printed box) to protect the hardware and also ensure that such hardware does not overheat, causing the computer unit to stop working in the worst situation. For this purpose, different models of custom 3D-printed boxes of different shapes and materials have been compared. In the experimental evaluation, identical stress tests have been carried out using different models of boxes. The results show that the physical design of the box greatly affects the temperature value reached in the sensor.

The remainder of this paper is organized as follows: first, Section 2 explores the main relevant related works in the field; then, Section 3 explains how the hardware of the WASN has been conceived, including: (i) the design and assembly of different commercial elements for each sensing node, (ii) the acoustic calibration process carried out to guarantee heterogeneity among nodes and (iii) the design process of 3-D printed boxes to protect the hardware. Next, Section 4 explains the physical and logical topology of the WASN, together with several tests and experiments that guarantee the correct functioning of the sensing nodes in a real-world environment. Section 5 details how should our approach be deployed in a new scenario. Section 6 discusses the resulting WASN, comparing it to other approaches. Finally, Section 7 concludes the paper, discusses the main findings of the work and explains the future work directions derived from this research.

## 2. State of the Art

Nowadays, in the literature, several designs of WASNs can be found, all of them with different strengths and weaknesses, depending on their goal and focus in terms of application. One can find networks for indoor localization purposes, or WASN to be deployed in a big city such as New York to evaluate the $L_{Aeq}$ and even try to detect certain pretrained events. In the recent years, there has been an increase in health concerns together with advances in sensing technologies. These have led WASNs to take a predominant place amongst the tools to survey around the acoustic health of the population living in urban areas [10], and also to monitor the biodiversity conservation in forests [11]. Not only in outdoor environments, but also at home, several WASNs have been tested to include networks on indoor environments to promote ageing at home with high quality of life [12] in the ambient assisted living paradigm, promoting active and healthy ageing at home. The advantage of WASN against other monitoring systems is that they respect the privacy of the users more than other technologies [13] (e.g., video surveillance), especially when the raw acoustic data are processed in the proper sensor, so that only labels travel to other nodes.

The number of connected nodes, as well as the distance and connectivity between them, is another of the issues that varies substantially depending on the application; finally, the computational capability of each of the nodes and the central server—if there is one—usually defines the requirements list of these kind of designs, especially when conducted using commercial hardware pieces. Several projects can be reviewed that have developed sensor networks that attend to different features and categories. In the context of the IDEA project [14], Domínguez et al. [15] propose the usage of low-cost nodes (with a cost of around EUR 50) to monitor outdoor environments that actively auto-check the frequency response of the microphone of each node by embedding a low-cost speaker that generates a periodical frequency sweep. Another example of an outdoor acoustic sensor network is the MESSAGE project, which is explained in [8]. In their work, Bell and Galatioto present the results obtained on a WASN of 50 nodes in which, apart from a noise detector module, each node incorporates traffic count and chemical sensor modules, using a microcontroller with low processing capabilities to do so. Regarding indoor WASNs, the homeSound project [1,12] proposes a network architecture with several sensing nodes that send their information to a concentrator node composed of a GPU with parallel computing capabilities, and later, the same team developed a new sensor with low-cost capabilities and more possibilities of real-time algorithm computing [16].

WASNs have been understood as a group of wireless microphone nodes spatially distributed over either an indoor or outdoor environment. The design has to take into account the scalability of the network, the delay of the acoustic signal, the synchronization of the nodes and the decision of where the computing of the data is conducted—if either in the cloud or locally in the sensors [17]. The low invasiveness of these systems made them more attractive for indoor applications, where patients or users are explicitly involved [18], especially when there is a clear intention of health monitoring or remote health tracking. Nevertheless, these sensors can also be used for surveillance applications when taking care of the elderly or disabled people [19]. In [18], the authors present an acoustic event detector system focused on a low-cost platform, recording and processing the sounds indoors.

The requirements of the latter applications both in indoor and outdoor environmental projects have led the priorities of the designs into three main issues: the low cost of the sensor design, the acoustic qualities of the designed sensor and the connectivity of the nodes, especially when there is no predetermined network to enable the link of all the nodes. Redundancy of sensors is one option to avoid false alarms in detection and improve the quality of the measurements collected. There is the idea of long-lasting networks, with lower maintenance, and in this sense, high quality—but low cost—microphones gather together good accuracy in the measurement stage and also lower incidences while working real-time. Finally, connectivity and ensuring that no information is lost in the data gathering to enable the central node to take decisions and activate warnings if needed is a crucial focus nowadays. In this sense, the reader can find an exhaustive review of the last trends of WASN design in [20]. Several examples can be found in [21] for the well-known project Sounds of New York [22,23], or [24], which maximizes network lifetime using coverage sets by means of designing its schedule.

After the work conducted by Alías et al., another contribution has appeared in the literature, which is worth mentioning for its similarity to the network proposed in this paper: Arce et al. [9] present a highly-scalable wireless acoustic sensor network that can monitor urban environments by recognizing a given set of sound events or classes. The nodes are based on Raspberry Pi (RPi) devices that do not only have the capability of recording the environmental sound but also to recognize different sound events by means of Convolutional Neural Networks (CNNs). Running the classification algorithms locally in the node has not modified substantially the results of accuracy compared to cloud-based solutions, proving that the local computation of deep learning is a good and feasible option for WASNs. Despite [9] having done all their tests in an outdoor environment, their proposal could be adapted for an indoor scenario. For this purpose, the connectivity would have to be reviewed and improved, in order to guarantee the coverage in all the rooms inside a house, a hospital, an university or a library (among others). The proposal of the WASN presented by Arce et. al. in [9] will be further compared to the approach presented in this paper to detail the essential contributions of the proposed WASN, especially orienting the comparison to three items: (i) low cost of the devices, (ii) acoustic quality of the microphone and raw data collection and (iii) ensured connectivity between nodes.

## 3. Sensor Design

Monitoring an acoustic environment requires a reasonable quantity of reliable acoustic sensors to gather samples of the acoustic landscape of the place. For these applications, the sensors should meet the following requirements:

1. **Continuous measuring:** each sensing node must be capable of measuring continuously and without being interrupted, to avoid sample loss.
2. **Maximum semblance between devices:** To obtain comparable metrics (in terms of measured noise level but also in terms of temperature response, speed and reliability), all the sensing nodes of the network should be as similar as possible among them. Additionally, they should be calibrated prior to their deployment, to ensure that the results that they are supplying are comparable.
3. **Low cost:** The price of each node of the network must be moderate to ensure the deployment of many sensors. This way, a wider area can be covered. For this work, a node is considered as low-cost if its price per unit is around EUR 100 or less, which is considerably low compared to Class-I commercial measuring sensors (which typically have a unitary price higher than EUR 1000 [25]).
4. **Scalability:** It is not only the price that limits the number of sensors that can be deployed over a certain area; other factors must be considered as well. For instance, adding nodes to the network should be as fast as possible, as human resources are usually needed for this task.
5. **Remote connectivity to access each node:** for monitoring purposes, addressing software failure or checking the status of the nodes, each node of the network should be accessible remotely. Occasionally, the physical location in which the sensors are deployed is not easily accessible to the technicians in charge of managing the sensing nodes. For this reason, remote access to the nodes becomes crucial when deploying a wireless network in a real-operation environment. However, it must be taken into account that despite having remote access to the nodes, there are problems that still must be solved physically, such as hardware failure (e.g., the microphone or the computing unit breaks and must be replaced).

The remainder of this section explains the different elements that compose the nodes of the proposed WASN and how have they been assembled.

### 3.1. Computing Unit of the Sensor

After an exhaustive evaluation of the available single-board computing units of the market, Raspberry Pi has been selected as the main computing platform for each individual node. Raspberry Pi meets all the requirements mentioned in Section 3 (ability to measure continuously, price lower than EUR 100, easy deployment and enables remote connection). A more exhaustive explanation on what platforms have been studied and discarded (such as Hummingboard [26], Cubieboard5 [27], Jaguar One [28], Banana Pi [29], PcDuino4 [30] or Beaglebone Black [31]) can be found in our preliminary work [16]. In general terms, these boards were discarded either due to their lack of a WiFi module, because they had features not required for this project (having, hence, a higher price) or because their online support community was not as big as the one offered by the chosen computational unit.

Among the different Raspberry Pi models, Raspberry Pi Model 4B (Broadcom BCM2711, Quad core Cortex-A72 (ARM v8) 64-bit SoC @ 1.5GHz) with 4 GB RAM has been selected. As the main storage unit, a 64 GB SD card containing Raspberry Pi OS Lite is used. This model allows the connection of peripherals (such as microphones) through USB 3.0 connectors. Moreover, as the board presents both wired (e.g., Ethernet) and wireless (e.g., WiFi or Bluetooth) connectivity, it offers flexibility for WASN deployments in different scenarios.

The capabilities of this board enable the performance of real-time signal processing [25]. To test to what extent the hardware is able of performing state-of-the-art acoustic signal processing, the board has been evaluated with a script that performs the following computations:

1. Acquires acoustic samples at a rate of 44,100 Hz with a bit depth of 16 bits.
2. Every 1 s, it calculates the Equivalent Loudness Level ($L_{eq}$) and calculates the spectrogram of the 1-second window audio.
3. After evaluating these two acoustic features, the spectrogram is used as an input of a Convolutional Neural Network (CNN) (i.e., MobileNet V2 [32], which occupies 8.8 MB of RAM) to perform acoustic event classification.

All the enumerated steps are carried out in less than 1 s using the selected Raspberry 4 model. MobileNet V2 has been selected as the classification algorithm as it is a CNN that has been specifically designed to be deployed in mobile or resource-constrained environments, as in the case of a WASN. Actually, MobileNets are architectures conceived for computer vision tasks that use depthwise separable convolutions, which are a type of convolutions lighter than regular convolutions. Depthwise separable convolutions split the computation into two steps: they first apply a single convolutional filter per each input channel (R, G, B) and then a pointwise 1 × 1 convolution to combine the output of the previous convolutions. This technique enables light-weight deep neural networks to be built [33]. In the case of spectrograms, as R, G and B channels do not have a relevant meaning except for visualization purposes, the spectrograms have been treated as greyscale images by replicating the same spectrogram to the three input channels, as typically done in the state-of-the-art audio classification tasks.

The main difference or novelty between MobileNet V2 compared to the first version of MobileNet is the incorporation of a type of layer named “inverted residual with linear bottleneck”. These types of layers enable us to use fewer parameters than the first version of MobileNet [32].

### 3.2. Microphone Selection and Test

Three models of USB electret microphones were initially considered. An advantage of these types of microphones—compared to other microphone types such as Micro Electro Mechanical systems (MEMS)—is that they do not require an external Analogue-to-Digital Converter (ADC). Moreover, as they are plug-and-play devices, they do not require the installation of drivers. These two facts together make the set-up of sensing nodes in a WASN easier, contributing to the scalability of the system.

The three specific models that were considered and evaluated are: Gyvazla (Gv) [34], Sandberg streamer model 126-19 (Sb) [35] and Saramonic SR-ULM10 [36]. Nevertheless, the SR-ULM10 was discarded because its gain cannot be adjusted, thus avoiding a proper calibration process, which is required in this prototype. These microphones were chosen based of technical specifications and cost per unit. While on the technical side the three models are comparable according to their manufacturers, the price per unit varies somewhat more. Specifically, the cost varies between EUR 10.99 and EUR 39. Furthermore, Gyvazla microphone is the cheapest and Saramonic SR-ULM10 the most expensive. In addition, the Sandberg streamer microphone model costs about EUR 29.

Two microphones of each model were compared to assess whether there are substantial differences between microphones of the same model. First, they were calibrated using a calibrator (Brüel and Kjaer 4231) emitting a 1 kHz tone at 94 dB. In order to avoid clipping, the sound card input gain for the Gyvazla microphones was adjusted through the *alsamixer*, which is a graphical mixer program that allows one to adjust the volume and set-up sound settings in Linux. Specifically, it was set to 60% (15.0 dB) for the Gv1, and to 77% (22.5 dB) for the Gv2. Conversely, the default input gain was used for the Sandberg microphones (100%, −2.0 dB). Then, a calibration factor was computed for each of the four microphones, (i.e., the number that the recorded waveform of the calibration tone should be multiplied by to obtain 94 dB). The obtained factors are shown in Table 1.

**Figure 1:**
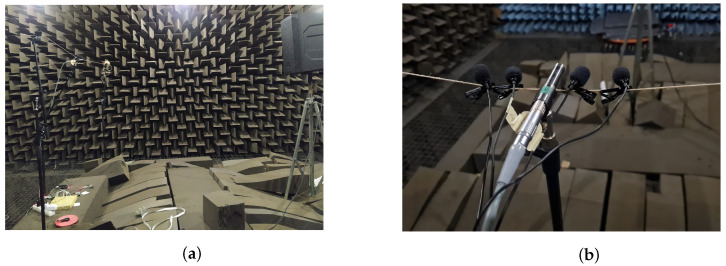
Set-up to measure the linearity of the two selected USB microphones. (**a**) Set-up with the speaker, the reference microphone and the evaluated microphones in the anechoic chamber. (**b**) Reference microphone (central microphone) and evaluated USB microphones (surrounding the central microphone) in the anechoic chamber.

After the calibration process, different measurements were made to evaluate the linearity of the microphones. To this end, a loudspeaker Electro-Voice (ELX200-10) was used to emit 1 kHz tones with different reference levels, from 50 dB to 99.2 dB, in 10 dB steps. The tested microphones together with a free-field reference microphone (G.R.A.S. 40 BF) were placed at 1.5 m from the loudspeaker. This distance was chosen following the ISO 3382-2 [37]. Testing the linearity of microphones is important to see how they respond regarding to the level to which they are exposed. This gives an idea of the reliability of the measured level, regardless of whether the microphone sensitivity has been adjusted using the typical 94 dB calibration tone at 1 kHz. The results of the test are shown in Table 2 and pictures to show the set-up in the anechoic chamber are shown in Figure 1.

If linearity performance shown in Table 2 is compared between the two models considering only the values above the background noise, the Sb microphone results in a better one. While the maximum deviation obtained with the Sb model is 1.4 dB (for 99.2 dB reference level), meaning that we measured 97.8 dB instead of 99.2 dB; for the Gv model, a deviation of 3 dB was measured (for 60 dB reference level), meaning that we measured 63 dB instead of 60 dB. If the results acquired between microphones of the same model are compared, levels obtained from Gv1 and Gv2 showed differences of about 2 dB for most of the reference levels. In contrast, the maximum difference between Sb1 and Sb2 is 0.6 dB only and just for one of the reference levels (99.2 dB). In addition, the background noise levels measured for the Sb model are noticeably lower than those measured for the Gv microphone. This background noise comes mainly from the electrical noise of the microphone together with the USB adapter. After analyzing the results listed in Table 2 along with the ease of the calibration process, Sandberg streamer microphone model has been chosen.

### 3.3. Sensor Enclosure

For the enclosure of the components, a 3D box has been designed. The reason to design a box to cover the sensing node is twofold. On the one hand, it protects the computing unit in case of falls. On the other hand, it protects it from undesired manual disconnections of the different components (such as the USB microphone, the SD card or the power supply) and, moreover, protects the user from touching the computing unit when it is working at elevated temperatures. However, designing an optimal box that enables protection without interfering with the ventilation and heat dissipation of the computing unit is not trivial. In this section, different designs together with their associated overheating problems and solutions are introduced, assuming that no extra ventilation and heat dissipation was desired due to its high interference with audio gathering, and extra consumption and cost.

To evaluate the temperature behavior of different set-ups, identical stress tests have been carried out under the same ambient temperature (21 °C) which has been verified using a DHT11 (manufactured by Sain Smart, Delaware, USA) [38] temperature sensor. The tests, which were conducted with an exhaustive software from the stressberry (pypi v0.3.3) [39], consisted of the following steps:

1. Measuring the temperature for 1 h without stressing the RPi (i.e., relax period).
2. Measuring the temperature for 2 h stressing the RPi (i.e., stress period).
3. Measuring the temperature for 1 h without stressing the RPi (i.e., second relax period).

**Figure 2:**
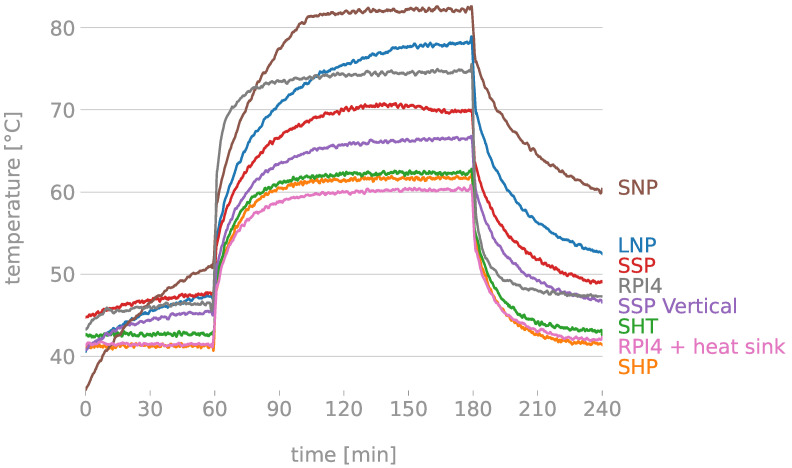
Temperature responses in time of the RPi using different setups (heat sink and enclosure) when performing a stress test.

Figure 2 shows the results of the stress tests conducted over the computing unit with different set-ups (i.e., with and without heat sinks and with different covering boxes).

**Figure 3:**
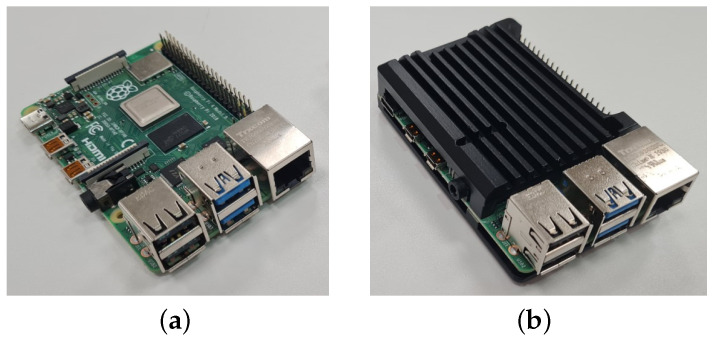
Raspberry Pi 4B without (**a**) and with (**b**) heat sink. (**a**) Raspberry Pi 4B without heat sink. (**b**) Raspberry Pi 4B with heat sink.

The temperature of a RPi 4B without any type of heat sink (i.e., naked device) has been taken as a reference (see Figure 3a).

It can be appreciated that the RPi reaches 75 °C when it is under stress. It suffers an increment of 54 °C compared to the ambient temperature. Furthermore, during the first relax time previous to the stress, it works at 45 °C and, after the stress, it practically reaches the 45 °C in an hour.

In order to help the RPi work under better conditions, an aluminium heat sink was placed on top of the RPi (see Figure 3b). This heat sink could not include a fan as it would affect the measurements of the microphones. As is known, the bigger the surface of the heat sink, the more dissipation it shows. For this, the selected heat sink covers all the Raspberry Pi. The results with this heat sink are shown in Figure 2. As it can be observed, it starts with 42 °C as baseline temperature when it is not under stress. Practically, it is the same initial temperature as when the RPi is naked. In the stress phase, it reaches 60 °C; which means an increment of 39 °C regarding the ambient temperature. After the stress, after an hour, it is capable of reaching back the 42 °C. Compared to the test where the RPi has no heat sink, the heat sink achieves a reduction of 15 °C when stressed.

Using 3D modelling with SketchUp, multiple boxes (exposed in Table 3) have been created. These boxes are meant to contain all the hardware inside. They have been printed using Polylactic Acid (PLA), known for its low thermal conduction (0.13–0.16 W/m·°C), which enables us to keep the enclosure cold and retain the temperature. All the boxes can be closed using a metric 4 (M4) screw.

**Figure 4:**
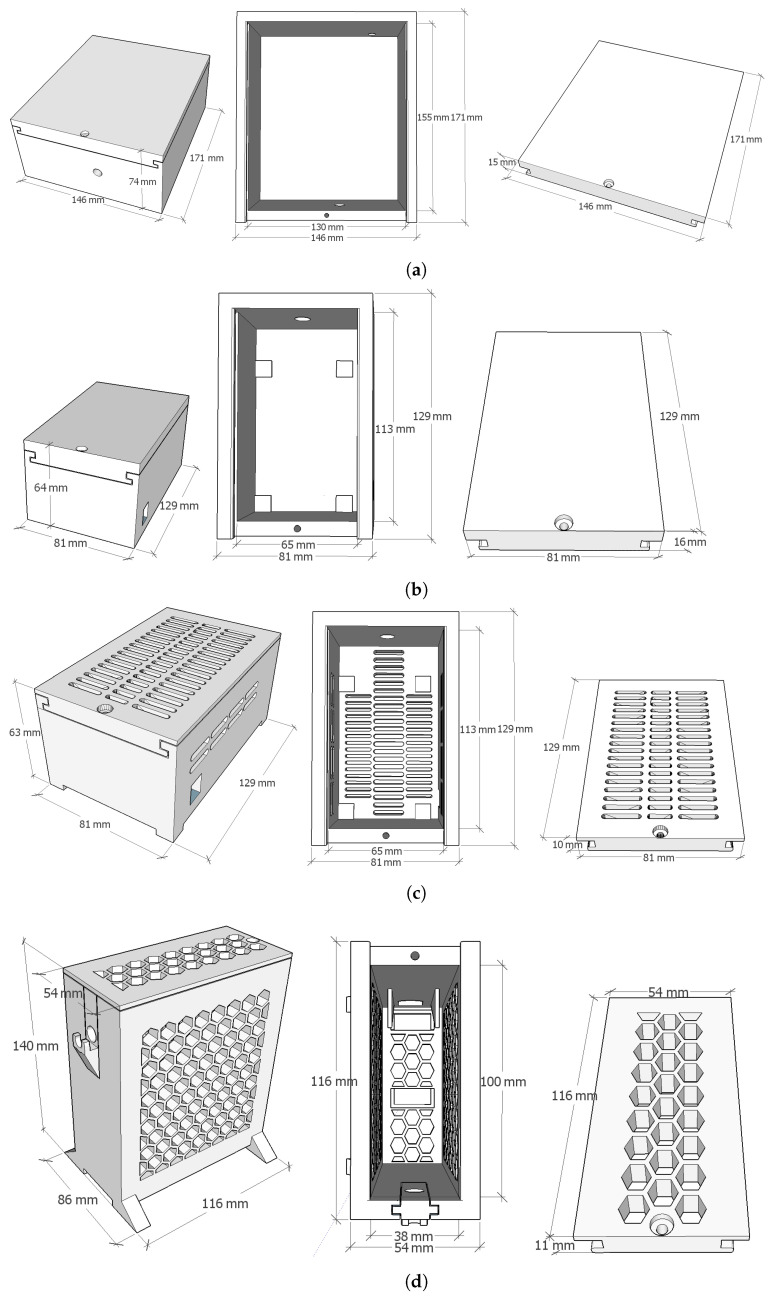
Boxes’ designs. Perspective view of the assembled box, top view of the base and perspective view of the cover. (**a**) Large Non-holed PLA box (LNP). (**b**) Small Non-holed PLA box (SNP). (**c**) Small Slot-holed PLA box (SSP). (**d**) Small Honeycomb-holed PLA box (SHP) or Small Honeycomb-holed TPU box (SHT).

The first created model had the shape of a completely hermetic box, LNP (see Figure 4a). This box would contain the RPi device and a power connector (such as a 5V mobile charger). It has two holes; one for the microphone and another one for the wire to power the RPi. Even though the box was functional, once printed, it was observed that the size of each sensing node would be too big to fit an indoor environment, which could lead to the perception of intrusion or obtrusion once deployed [3].

Aiming to reduce the dimensions of the box, the SNP (see Figure 4b) box was created. In this second approach, the connector to power the RPi was placed outside the box. This way, a wire would be connected directly to the RPi port. Thanks to these, the box contains only the RPi device, which enables us to reduce its dimensions considerably (from 146 mm × 171 mm × 74 mm to 81 mm × 129 mm × 64 mm). However, as it can be observed in Figure 2, this box obtained worse thermal conditions for the RPi. For this reason, aiming to reduce the temperature of the RPi, the SSP box (see Figure 4c) was designed. It has the same design as the SNP box, but the cover is thinner and slot-holed. As the air flows upwards when it is warmed, the holes are placed in the highest part of the box. Also, there are holes at the base to enable the entrance of fresh air.

After this third design, some temperature tests revealed that the RPi has better temperature conditions if it works vertically. Therefore, the SHP (see Figure 4d) was created containing the RPi placed vertically inside the box. This box is honeycomb-holed in order to avoid using supports while 3D printing and, therefore, avoid useless PLA and faster printing. Furthermore, in this design, the support for the microphone is changeable, as we noticed that printing it with Thermoplastic Polyurethane (TPU) material allows to better adapt to the microphone to the box, compared with the same support printed with PLA. This is thanks to the flexibility of the TPU.

The last box model is SHT (see Figure 4d), printed using a TPU, which is a thermally conductive, electrically insulating plastic model Ice9${}^{\mathrm{TM}}$ Flex (4 W/m·°C) from TCPOLY [40]. Our original hypothesis was that it would help to dissipate the temperature from the RPi due to its thermally conductive properties. However, as shown in Figure 2, it has worse results than the one printed with PLA.

Figure 2 illustrates the temperature of the RPi out and inside the boxes. As shown, the SNP box (brown line) obtains the worst results, followed by the LNP box (navy line). Actually, these two boxes reach higher temperatures than the naked RPi (grey line). This is due to the lack of holes in the box, causing a lack of proper ventilation. The SSP box (red line) is the first design that obtains lower temperatures than the naked RPi. Therefore, we appreciate that the effect of holing the cover is important.

Additionally, the purple line illustrates the effect of using the same box (i.e., SSP box), but placed vertically. Results show that the RPi temperature decreases about by 5 °C. This way, authors designed the SHP box, which allows a better placement of the RPI and more ventilation.

The last three lines of the graph obtain similar results: the green line belongs to SHT, the orange line belongs to SHP and the pink line belongs to the RPi with the heat sink and without any type of coverage. As can be appreciated, the effect of the boxes SHT and SHP is almost negligible, as the temperature when protecting the RPi with these two designs remains almost the same.

To conclude with this section, we have demonstrated that the best thermal conduction box is the Small Honeycomb-holed. Regarding the building materials of the boxes, to choose between using PLA or TPU from TCPOLY [40], authors considered that PLA is the cheapest option (1 kg costs EUR 10–20, in front of EUR 203.88 of 1 kg of the TPU from TCPOLY) and obtains practically the same results as if the RPi was with just the heat sink outside of a box; with a difference of 2 °C only. For all these, the selected box is SHP.

### 3.4. Assembly of the Nodes

After the different components of the WASN nodes were selected, each node was assembled to contain:

- **Single-board Computer:** a Raspberry Pi Model 4B of 4 GB RAM with an SD card of 64GB and a heat sink.
- **Microphone:** An omnidirectional electret condenser microphone [35] with a flat frequency response between 50–18,000 Hz, a maximum sampling rate of 96 kHz/24 bit and a Signal-to-Noise ratio (SNR) of 84 dB. The microphone is connected to the Single-board computer using a USB 3.0 port.
- **A 3D-printed designed box:** The box integrates the microphone sensor and the computing unit into a single element. Additionally, it offers protection against falls or undesired manual disconnections of the elements that compose the sensor.
- **Power supply:** a 5V-3A charger with USB-C connector powers the system.

**Figure 5:**
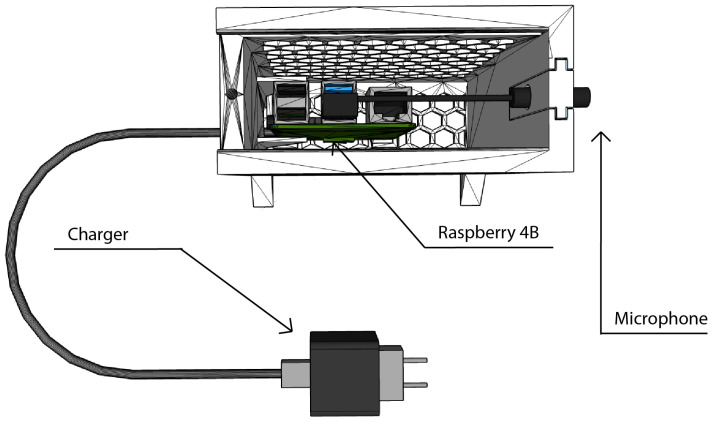
Conceptual integration of the hardware elements of the sensor. The 3D model of the Raspberry Pi has been retrieved from [41].

Figure 5 represents a sketch of the integration of the elements that compose each node as a low-cost sensor prototype. The characteristics of the protection box make this sensor suitable for indoor environments.

## 4. WASN Design

Once all the nodes are assembled and integrated into their boxes, there are two types of network topologies that should be designed prior to the deployment of the network to allow connectivity between devices. The first one is the physical topology, which refers to the physical location of nodes in the scenario that is aimed to be sensed. This topology will be dependant on the specific location where the network is to be deployed. The second one is the logical topology, which refers to the architecture of the communication mechanism for all nodes in a network. If all the potential scenarios are similar in terms of size and distribution, this topology can be shared between different scenarios. This section explains the design of these two types of topologies for the proposed WASN using a real-world scenario.

**Figure 6:**
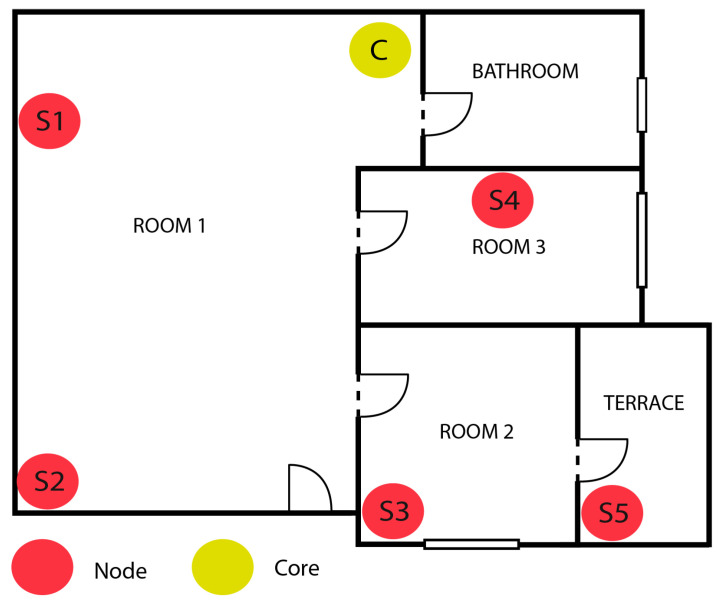
Example of WASN deployment in an indoor space.

Real-time acoustic mapping, which is one of the many purposes that a WASN could have, has been selected as a tool to evaluate the proposed approach. These maps are tools to monitor remotely the amount of sound present at different points of a specific environment. The scenario has been tested with the proposed WASN for a relatively long period (about 1 year and 3 months). The main goal of the experiment was to monitor an area composed of three rooms and a terrace. A sketch map of the area in which the WASN was deployed is shown in Figure 6. In this example, the bathroom was not monitored due to privacy and sensor proximity to water and humidity.

### 4.1. Physical Topology

To design the physical topology of the network (i.e., where the acoustic nodes will be located), it must be considered that in large spaces—more than 10 m^2^ scenarios (e.g., room 1 in Figure 6)—several sensors are required, as different areas can generate diverse pressure levels. In this case, at least one sensor per activity area (where an activity area refers to a part of the room that usually produces a similar and meaningful type of sound, such as a television or a piano) in the room is required for correctly mapping the sound in contrast of a small space (e.g., rooms 2 or 3 in Figure 6) where one microphone is enough to capture a representative value of the sound level of the room. Although the microphone pattern of the proposed sensor is omnidirectional, given that the node and microphone are mounted in the protection box, it is recommended to position the microphone in corners or walls targeting the sound activity zone.

Despite having tested the prototype in a single location, the design of the Wireless Acoustic Sensor Network is invariant to the deployment characteristics. The only part that is dependent on the environment is the final location of nodes in the areas to be monitored. The design of the WASN consists of:

- Nodes: *N* number of sensors identical to the ones described in Section 3.4, located in strategical places to sense the environment. In the proposed prototype, uninterruptedly, each node sends the acoustical level measured over a programmable period of time to the core of the network.
- Core: A single Raspberry Pi 4B per network that acts as a gateway. The purpose of this node is to receive data from all the synchronized nodes and upload them to the cloud, where a graphic representation of the acoustic map could be visualized. Centralizing the nodes allows fast remote management accessing through the core. It is important to place the core next to the router of the indoor space and do a wired connection to it to minimize connectivity problems. This node does not compute any measurement by itself, and it does not need any microphone.

An optimized database for Internet of Things (IoT) is required to save all the nodes’ data in each programmable period. A potential IoT platform could be Thingspeak, implemented and used in this project, which is an IoT analytics platform service that allows the user to aggregate, visualize and analyze live data streams in the cloud [42].

### 4.2. Logical Topology

As explained in Section 4.1, nodes send data to the core node. As wireless modules, RPi includes Bluetooth and WiFi 2.4 GHz and 5 GHz modules. To choose one or another, the distances and difficulties different nodes may face in order to send the data must be considered. As this work focuses on indoor environments, these difficulties will be long distances and walls of different materials and thicknesses. Taking that in mind, WiFi has been selected due to its bigger range of connectivity compared to the Bluetooth module integrated into the RPi. Additionally, the 2.4 GHz band has been chosen over the 5 GHz one, given that the connectivity range from the first one is 46 m (150 feet) in front of the 15 m (50 feet) offered by the second one. These distances are valid for indoor environments [43].

There are two ways to send data to the core:

- Direct connection: Each node connects directly to the core and sends its data.
- Using other nodes: A node uses the connectivity of another node to send its data. This topology is called a mesh connectivity [44]. With *n* being the number of devices in a mesh network, then each device must be connected to n−1 number of devices. The total number of links can be calculated using the following equation:
  (1) $$\mathrm{Total}\;\mathrm{number}\;\mathrm{of}\;\mathrm{links},\;L=\frac{n\cdot (n-1)}{2}$$
  In such networking, the nodes can create and update their links automatically. Therefore, in case a route to a node becomes disabled, the network will automatically rebuild a new route through another radio node so that the information can still reach its destination [45].

The connectivity performance of the WASN was tested according to the following configuration. The nodes were programmed to compute the sound equivalent level ($L_{eq}$) every 20 s. Equation (2) shows how to calculate the $L_{eq}$. In the equation, $L_{eq}$ represents the sound equivalent level (in dBs), $p_{0}$ is the reference pressure level (which was set to 20 μPa), $p_{A}$ are the samples of the measured pressure levels, and *N* are the number of samples—which depend on the sampling rate and the amount of time of equivalent level to be calculated.

Once the $L_{eq}$ values of 20 s were calculated in each node of the WASN, the level (in dB) was sent to the core. Then, the core aggregated the values from the five nodes and sent them to the thingspeak cloud platform.

(2) $$L_{eq}=10\cdot \log\left[\frac{1}{N}\cdot \sum_{n=0}^{n=N}\frac{{\left(p_{A}\left(n\right)\right)}^{2}}{{\left(p_{0}\right)}^{2}}\right]$$

A first deployment of the WASN was carried out following the direct connection strategy. In this strategy, all the nodes send their data directly to the core, without intermediate steps.

**Figure 7:**
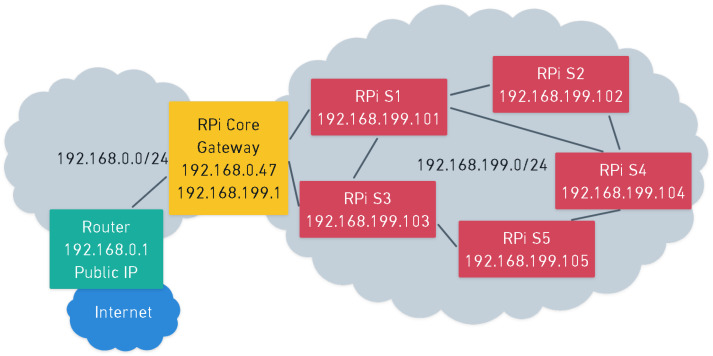
Example of a logical network design with five nodes (in red), one core (in yellow) and one router (in green).

Due to the architectonic features of the scenario in which the system was deployed, some frames were lost when using this approach, especially the nodes S4 and S5, which are the ones that are physically located further from the core. Moreover, the connection with the node S5 was lost after 12 days of operation. Therefore, a mesh connection was implemented. We used [46] to configure such a mesh in the RPis. Figure 7 shows a schematic of the logical network configuration. As it can be seen, the mesh uses the network 192.168.199.0/24 where each node has the IP 192.168.199.X, where X represents the node number plus 100. On the other hand, the core has the IP 192.168.199.1 inside the meshed network.

On the private network created by the router, the core has a DHCP IP (in Figure 7, 192.168.0.47). This network (192.168.0.0/24) contains the rest of the devices (such as computers, smartphones or tablets). Therefore, the mesh network is only available and used by the nodes of the WASN (i.e., RPis). In case a device outside the meshed network wants to communicate with a RPi inside the meshed network, it has to do it through the core device.

When a node needs to send data to the core, it will jump from one node to another one as many times as needed to reach the core. For instance, in Figure 7, S4 does not have direct connection to the core, but it can reach the core using multiple paths, the fastest ones being the following four: 1. S4-S2-S1-Core 2. S4-S5-S3-Core 3. S4-S1-S3-Core 4. S4-S1-Core

The fastest path is the fourth option, as it only has two jumps to reach the core. The third one will probably not be used, as S1 will receive the data and will send them directly to the core. Although, in case S1 does not have connectivity to the core, it can send the data to S3. Once the core receives the data, it will forward it to the router and, lastly, the router will forward it to the Internet.

The percentage of frames received by the platform during the experimental evaluation in the real-world deployment is shown in Table 4. It can be observed that the mesh strategy achieves better results (Table 4) than the direct connection, with a percentage of received frames higher than 98% for S5 and higher than 99% for the other nodes. Nevertheless, the connection with S5 was lost after 9 days of operation. To solve that issue, a weekly reboot of S5 was programmed using *crontab*. This way, the loss of connectivity of S5 was avoided and a percentage of 99% was achieved as it can be observed in the third row of Table 4.

## 5. WASN Deployment Methodology

This section details the steps that must be followed for the installation of the proposed WASN once the analysis of the area to be monitored has been carried out and the position and number of acoustic nodes have been determined. Specifically, this section will explain how to (1) initially deploy an acoustic wireless sensor network and (2) add more nodes to an active system.

### 5.1. Installation of the Core Node

As the core acts as the gateway between all the sensor nodes of the local area network and the Internet, it has to be configured first to establish the connection to the rest of the sensors in a later time. To do so, the next steps must be followed:

1. **Core assembly:** First of all, the heat sink must be integrated to the Rpi. Then, the Raspberry must be placed in the designed box. As this is the core node and will only be used as a gateway, this node will not need a microphone.
2. **Core wiring:** the Raspberry should next be plugged into a power source near the router to enable an Ethernet connection between the Raspberry and the router.
3. **Connection to Ethernet:** Once done, an Ethernet wire should be passed through a hole in the cover of the box and, next, must be connected to the RPi. The box can be closed with a M4 screw.
4. **Connection to the mesh:** The core must, then, create a private network and connect to it through WiFi (following the steps of [46]). Additionally, a static IP must be configured, enabling the nodes to send data to it.
5. **Connection to an external database:** The core node must be configured to send processed data to an external database (e.g., Thingspeak). The bytes sent to the cloud would depend on the application. For the proposed project in this paper (i.e., the generation of a noise map), the bytes to be sent would be the $L_{eq}$ levels calculated in the rest of the nodes and a timestamp.

### 5.2. Installation of New Nodes in an Active System

The process to add nodes to the acoustical network is systematic and can be done whenever a new sensor is required. Note that the system needs a minimum of one node to work. Steps to add a node are described below:

1. **Node assembly:** Place the heat sink on top of the Raspberry, connect the microphone to the central unit and put both components inside the box. The assembly must look as it does in Figure 5.
2. **Node wiring:** Plug the node to the power source. The box can now be closed with a M4 screw.
3. **Static IP configuration:** connect the running node to a computer using an Ethernet cable to set up the WiFi mesh with an unused IP and static DHCP and obtain the WiFi MAC address (following the steps of [46]).
4. **Update the core node:** add the MAC address of the node to the configuration file of the core so it recognizes the new node as part of the mesh using its MAC.
5. **Microphone calibration:** calibrate the microphone to 1 kHz, 94 dB and set the calibration factor to obtain a referenced sound pressure level.

## 6. Discussion

This section aims at comparing the proposed approach to WASN designed in other works. For this purpose, Table 5 illustrates the main differences between the sensing nodes between three big research projects (FIWARE-based WASN [9], SONYC [23] and MESSAGE [8]) and the approach presented by the authors, first in the previous work of [16] and in the present work.

### 6.1. Microphone

The type of microphone mostly used for acoustical WASN is condenser or condenser-electret according with the comparison made in Table 6. The microphone integrated in the presented work increases the frequency response from previous prototypes [16] and enables to obtain more frequency resolution for event detection algorithms meanwhile has less deviation in SPL measurements. It is observed in Table 6 that the author proposal is the only plug-and-play electret microphone that offers calibrated SPL values and has a lower, price with a cost of EUR 29, compared to the other plug-and-play microphone [9] with a significant cost of EUR 140 as of September 2022. In addition, if the sizes of these two microphones is compared, the one selected in this work is significantly smaller, resulting in a smaller sensor. Another aspect to take into account is the signal-to-noise ratio (SNR) of the microphones. Furthermore, the SNR obtained by the proposed microphone is low enough so as not to mask the sounds to be monitored. Even though MEMS microphone technology has improved over the year, one of the main problems with this typology of microphones is the electrical background noise that they generate (see [47]).

### 6.2. Connectivity

Regarding connectivity, it is worth mentioning that our previous approach [16] and the approach presented in this work are the only ones from the table designed to be deployed in indoor environments. The rest of the approaches were designed for outdoor WASNs.

As it can be observed in the table, MESSAGE [8] is the only one that uses ZigBee to connect the nodes. Such connectivity protocol can also be used for indoor spaces. However, adding such a protocol involves using an external antenna, which would increase the cost of the proposed nodes. Moreover, ZigBee is a widely used protocol in indoor spaces (mainly in home automation), but each maker can use it in different ways, causing incompatibilities among them. Furthermore, ZigBee has a 256 Kbps throughput, compared to the 600/54 Mbps throughput that can be achieved using WiFi [48]. For these reasons, authors consider WiFi a better option, as it is integrated into the Raspberry Pi computing unit. This way, no additional costs per node are required. Considering that there is a linear relation between the cost of the nodes and the final cost of the WASN, reducing costs per node is of great importance in terms of scalability. Additionally, the selected connectivity technology (WiFi) enables achieving faster throughput, which is beneficial for real-time applications.

Regarding the comparison of direct WiFi connections to meshed connections, Table 4 has demonstrated that there is a better performance when using a mesh network, compared to a direct WiFi connection. Therefore, the presented meshed approach is more suitable for the proposed application than the approaches designed for the SONYC project [23] and our previous approach [16].

The work presented by Arce et al. [9] uses a mesh connection as well. In this case, they use the Optimized Link State Routing (OLSR) protocol. In the presented approach, we chose to use the Hybrid Wireless Mesh Protocol (HWMP). Both approaches are standard and widely used mesh protocols [49]. As it can be seen in Table 4, the HWMP protocol has enabled us to obtain good results as all the nodes of the network were able to send data frames with more than 99% of success. As the HWMP protocol was working correctly, no other mesh protocols were experimentally evaluated.

### 6.3. Computing Unit

As can be observed in the table, the work that differs more in terms of computing unit is the MESSAGE approach, which uses a light PIC microcontroller instead of a single-board computer as the Raspberry Pi or the Tronsmart. In this sense, although the MESSAGE computer platform is the one that offers the cheapest computing unit, it would be unable of performing expensive (in terms of hardware) computations in the edge such as acoustic event classification using machine learning or deep learning approaches using its 64 KBytes of program memory and 3968 Bytes of data memory. Considering that the scope of the MESSAGE project was to measure noise levels instead of performing acoustic event classification in the edge, the usage of such a light computing unit was justified for their approach, but is not sufficient for the scope of the current paper.

Raspberry Pi model 3B, chosen by the FIWARE-based platform [9], enables the performance of acoustic signal processing and classification in the edge. Actually, in previous works (i.e., [16]), the authors of the current paper also chose to use a Raspberry Pi model 3B+, which is a very similar on-board computer. The main difference is that while the processor of the first one operates at 1.2 GHz, the processor of the latter one operates at 1.4 GHz. However, after performing several classification tests, we realized that upgrading the computing unit to a newer model (Raspberry Pi model 4B with 4GB of RAM) allowed us to obtain 50% faster classification results when using state-of-the-art deep neural networks (i.e., MobileNet V2 [32], which has a size of 8.8 MB) as classification algorithms, which enabled us to reduce the acoustic window. Actually, it took 1.3 s to perform the classification of a 4 s fragment in a Raspberry Pi Model 3B+ and 0.7 s to do the same action using the same algorithm in a Raspberry Pi Model 4B. These timings correspond to an algorithm that, from a 4 s raw audio fragment, calculates the spectrogram of the audio file, normalizes it and passes it through a MobileNet V2 convolutional neural network stacked with a decision tree. More details regarding the algorithms can be found in [25].

Compared to the computing unit used in SONYC [23], the Tronsmart MK908ii device has 2 GB of RAM, compared to the 4GB of the Raspberry Model 4B used in this project. For this reason, authors believe that the computing unit presented in this paper (Raspberry Pi Model 4B with 4GB of RAM) is the most suitable for a real-time acoustic event classification application.

### 6.4. Power

Regarding the powering of the system, although indeed the battery-based systems can be physically deployed at almost any location, having to charge or replace the battery is not convenient for a long-term deployment. This issue is emphasized when thinking about the scalability of the system. For this reason, we think that the best way of powering a static device is to connect it to a standard plug. In the proposed approach, the plug could be replaced by a standard 5 V–3 A power bank if mobility was required, but due to the need for a human interaction for charging it periodically, this powering system is not recommended.

## 7. Conclusions

This paper proposes the design of an indoor WASN with distributed computational capability to face several ambient assisted living challenges. The work has three main focuses in its design: (1) the individual nodes of the network, which are composed of a Raspberry Pi computing unit and a USB microphone; (2) the interconnection of the different nodes among them using a meshed connectivity; and (3) the design of a box to cover and protect the hardware while preventing it from overheating.

The final prototype designed satisfies the requirements initially described, and gives the team wide research options to work in indoor environments, with coverage in several different spaces, and with the deployment of machine learning algorithms to detect acoustic events in real-time. The price of the design is affordable and in the criteria of low-cost, what gives it the opportunity of having a large number of devices per home if necessary. The connectivity is solved even in environments with low WiFi coverage, and the microphones used have the required quality to detect events and measure $L_{eq}$ to draw the sound map, as the use case presented in this work. Furthermore, the microphone study and analysis has been conducted with more precision and detail than most of the solutions proposed in the literature for low-cost WASN, where the acoustic quality of the sensor was barely taken into account, prioritizing its price and assuming a change of the device for a bad performance.

Future work directions involve replicating the experiments exposed in this paper in multiple indoor environments with different architectural distributions to validate that the meshed topology is valid for different locations as long as the distances explained in Section 4 are respected. In this line, it is important to work in order to achieve the redundancy needed for the nodes that are farther from the core, i.e., if it only has a connection to one node and this node stops working, this failure will also have an affect on a node that works. However, it is important to make a good selection of the placement of each node and to assure that redundant nodes in terms of sound evaluation—in this example—and connectivity are not used, as it will clearly affect the total cost of the WASN. The more nodes needed to survey an area, the more expensive the WASN will be. To say more, in the case of big spaces and, depending on the needs of sound event detection, these can be places where with one or two nodes per floor are enough to cover the sound topology; if the application was in a factory, for instance. In these situations, the WiFi coverage could not be enough and other type of wireless protocol should be considered for usage (e.g., Zigbee for medium spaces or Long Range Modulation (LoRa) for huge spaces and buildings). The costs of displacing various nodes with WiFi in front of fewer nodes with another type of wireless protocol should also be considered. Or, instead of adding a completely new wireless protocol to all the nodes, using it only in the farthest nodes could also be a solution.

On the other hand, authors also want to discuss the algorithm that can be run in each node. The placement of each node must be taken into account when using one algorithm or another, because not all the rooms are used for the same purposes. To illustrate, in an industry, there can be a room with different types of machines, another room with people working and so on. In a closer example, in an ambient assisted living environment, such as the example shown in this work, the sounds coming from the kitchen are clearly different from the sounds coming from the bedroom. Therefore, each node algorithm must be trained with the specific types of sounds that can be found in the node room. Further study should be taken in this direction, as not every algorithm is valid for all types of sound. Some algorithms work better with sounds that are punctual and others with prolonged sounds.

Further microphone study is required. We showed the linearity of the microphones studied in Table 2, but the temporary drift of the microphones should also be considered. This drift is quite important as can affect to the computation of equivalent levels. In this sense, another relevant issue to study is the changes in the acoustic event detection accuracy depending on the quality and the deterioration of the low-cost microphones.

Last but not least, we propose to improve the evaluation of the performance of the network in terms of, in this example, acoustic event detection. A deep analysis of the algorithms implemented to compute in each node has to be conducted, both in terms of computational cost (already done in a preliminary study for this work), and in terms of accuracy and recall. For this purpose, the final goal of the acoustic event detection has to be set; e.g., the detection of activity in a flat, the detection of cooking in a house, the detection of people waking up in the morning and going to bed at night. Several possible—and powerful—applications in terms of ambient assisted living have already been tested by this team in the past [1,12,50], some of them more focused on the algorithm design and others more centered in the network design and distributed computing structure.

## Figures and Tables

**Table 1:** Calibration factors obtained for the different microphones.

Gv1	Gv2	Sb1	Sb2
1.922717	1.733560	2.103515	1.911244

**Table 2:** Microphones linearity measurements.

	Reference Signal Level	Tone at 1 kHz						
	**Background Noise**	**50 dB**	**60 dB**	**64 dB**	**74 dB**	**84 dB**	**94 dB**	**99.2 dB**
Gv1	57.2	58.3	63.0	66.2	75.7	85.6	95.6	98.2
Gv2	57.0	57.5	61.7	64.9	74.3	84.2	94.2	97.2
Sb1	34.6	49.7	60.1	64.1	74.1	84.2	94.1	98.4
Sb2	36.0	49.5	60.0	64.0	74.0	84.0	94.0	97.8

**Table 3:** Name of the created boxes, printing material, dimensions and the acronym used to identify them.

Box	Material	Dimensions (X × Y × Z)	Acronym	Figure
Large Non-holed	PLA	146 × 171 × 74 mm	LNP	Figure 4a
Small Non-holed	PLA	81 × 129 × 64 mm	SNP	Figure 4b
Small Slot-holed	PLA	81 × 129 × 63 mm	SSP	Figure 4c
Small Honeycomb-holed	PLA	86 × 116 × 140 mm	SHP	Figure 4d
Small Honeycomb-holed	TPU	86 × 116 × 140 mm	SHT	Figure 4d

**Table 4:** Percentage of frames received by the Thingspeak platform from the nodes (S1–S5) of the WASN. The first row corresponds to the percentages obtained with a direct connection between the nodes and the cores. The second row depicts the values with the mesh connection and the third row shows the percentages achieved when a weekly reboot of S5 was programmed. The values in parentheses correspond to the number of days sensor S5 sent data before losing the connection.

	Days	Frames Received in Thingspeak (%)				
		S1	S2	S3	S4	S5
Direct WiFi	199	98.26	97.42	99.27	86.25	76.97 (12 d)
WiFi mesh	90	99.37	99.22	99.95	99.95	98.28 (9 d)
WiFi mesh + reboot S5	40	99.97	99.95	99.81	99.87	99.07

**Table 5:** Comparative table of different WASNs. The last row represents the approach proposed in this paper. The last column indicates the price per node of each node of the network. Unavailable (N.A.) or missing prices have not been included in the table.

WASN	Microphone	Connectivity	Computing Unit	Power	Price
MESSAGE	Condenser	ZigBee	Microcontroller	D Battery or	N.A.
2013 [8]	microphone	+ GPRS	PIC18F4620	external power	
			(around EUR 5)	connection	
SONYC	Custom	WiFi direct	Tronsmart	120 V outlet	EUR 81
2017 [23]	MEMS	connection	MK908ii	+ PSU	
		to router	(EUR 50)		
P. Arce et al.,	miniDSP	WiFi mesh	Raspberry Pi	Battery	EUR 191
2021 [9]	UMIK-1	OLSR	3B	(EUR 20)	
	(EUR 140)	protocol	(EUR 31)		
Ginovart et al.	LYM00002	WiFi direct	Raspberry Pi	5 V–3 A	EUR 78
approach	(EUR 11)	connection	3B+	charger	
2021 [16]		to router	(EUR 37)	(EUR 12)	
Proposed	Sandberg	WiFi mesh	Raspberry Pi	5 V–3 A	EUR 120
approach	126-19	HWMP	4B-4GB	charger	
2022	(EUR 29)	protocol	(EUR 64)	(EUR 12)	

**Table 6:** Comparative table of different microphones implemented in each WASN. The last row represents the approach proposed in this paper.

WASN	Microphone	Frequency Response	Calibration	Plug and Play
MESSAGE 2013 [8]	Condenser	20 Hz–20 kHz	Yes	No
SONYC 2017 [23]	MEMS	10 Hz–10 kHz	Yes	No
P. Arce et al., 2021 [9]	Electret	20 Hz–20 kHz	No	Yes
Ginovart et al., 2021 [16]	Condenser	20 Hz–16 kHz	Yes	Yes
Proposed approach	Electret	50 Hz–18 kHz	Yes	Yes

## Data Availability

Data available upon request.

## Abbreviations

The following abbreviations are used in this manuscript:

3D	Three Dimensional
ADC	Analogue-to-Digital Converter
CNN	Convolutional Neural Network
CPU	Central Processing Unit
DHCP	Dynamic Host Configuration Protocol
HWMP	Hybrid Wireless Mesh Protocol
IoT	Internet of Things
IP	Internet Protocol
ISP	Internet Services Providers
LAN	Local Area Network
$L_{eq}$	Equivalent Loudness level
LoRa	Long Range Modulation
M4	Metric 4
MAC	Media Access Control
MEMS	Micro Electro Mechanical systems
OLSR	Optimized Link State Routing
PDR	Packet Delivery Ratio
PLA	Polylactic Acid
PSU	Power Supply Unit
RPi	Raspberry Pi
SD	Secure Digital
SNR	Signal-to-Noise Ratio
TCPOLY	Thermally Conductive Polymer
TPU	Thermoplastic Polyurethane
USB	Universal Serial Bus
WASN	Wireless Acoustic Sensor Network
WiFi	Wireless Fidelity
